# FL-DSFA: Securing RPL-Based IoT Networks against Selective Forwarding Attacks Using Federated Learning

**DOI:** 10.3390/s24175834

**Published:** 2024-09-08

**Authors:** Rabia Khan, Noshina Tariq, Muhammad Ashraf, Farrukh Aslam Khan, Saira Shafi, Aftab Ali

**Affiliations:** 1 Department of Avionics Engineering, Air University, Islamabad 44000, Pakistan; 211868@students.au.edu.pk (R.K.); noshina.tariq@mail.au.edu.pk (N.T.); 211862@students.au.edu.pk (S.S.); 2 School of Electrical Engineering and Computer Sciences, National University of Sciences and Technology, Islamabad 44000, Pakistan; muhammad.ashraf@seecs.edu.pk; 3 Center of Excellence in Information Assurance, King Saud University, Riyadh 11653, Saudi Arabia; fakhan@ksu.edu.sa; 4 School of Computing, Ulster University, Belfast BT15 1ED, UK

**Keywords:** Internet of Things (IoT), Linear Discriminant Analysis (LDA), IoT Routing Attack Dataset (IRAD), Hello Food (HF), Decreased Rank (DR), federated learning, deep learning

## Abstract

The Internet of Things (IoT) is a significant technological advancement that allows for seamless device integration and data flow. The development of the IoT has led to the emergence of several solutions in various sectors. However, rapid popularization also has its challenges, and one of the most serious challenges is the security of the IoT. Security is a major concern, particularly routing attacks in the core network, which may cause severe damage due to information loss. Routing Protocol for Low-Power and Lossy Networks (RPL), a routing protocol used for IoT devices, is faced with selective forwarding attacks. In this paper, we present a federated learning-based detection technique for detecting selective forwarding attacks, termed FL-DSFA. A lightweight model involving the IoT Routing Attack Dataset (IRAD), which comprises Hello Flood (HF), Decreased Rank (DR), and Version Number (VN), is used in this technique to increase the detection efficiency. The attacks on IoT threaten the security of the IoT system since they mainly focus on essential elements of RPL. The components include control messages, routing topologies, repair procedures, and resources within sensor networks. Binary classification approaches have been used to assess the training efficiency of the proposed model. The training step includes the implementation of machine learning algorithms, including logistic regression (LR), K-nearest neighbors (KNN), support vector machine (SVM), and naive Bayes (NB). The comparative analysis illustrates that this study, with SVM and KNN classifiers, exhibits the highest accuracy during training and achieves the most efficient runtime performance. The proposed system demonstrates exceptional performance, achieving a prediction precision of 97.50%, an accuracy of 95%, a recall rate of 98.33%, and an F1 score of 97.01%. It outperforms the current leading research in this field, with its classification results, scalability, and enhanced privacy.

## 1. Introduction

The Internet of Things (IoT) has brought about a profound transformation, enabling devices to connect and communicate with one another, making data sharing a reality [1]. This network of devices has contributed to the progress of several industries, including healthcare and transportation, by establishing a synergistic environment where gadgets collaborate to collect, analyze, and share information [2]. The ability of IoT to change and smooth certain aspects of our day-to-day operations makes IoT significant [3]. The IoT has become an essential element of modern life, involved in many aspects such as homes, cities, automation, and healthcare [4,5]. The unparalleled capacity of this technology to enhance efficiency, productivity, and overall quality of life is incomparable [6]. At its foundation, IoT represents a fundamental paradigm change, where everyday objects are empowered with the capacity to connect, communicate, and collaborate effortlessly [7]. This interconnectedness offers extraordinary prospects for efficiency, productivity, and convenience across multiple disciplines. IoT allows for remote patient monitoring, individualized treatment plans, and predictive analytics in healthcare, eventually boosting healthcare outcomes and saving costs [8]. IoT provides intelligent infrastructure management, efficient resource allocation, and increased public safety in smart cities through real-time data insights [9,10]. IoT revolutionizes industrial processes by allowing for predictive maintenance, supply chain optimization, and intelligent manufacturing, generating significant increases in operational efficiency and cost reductions [11,12]. In addition, IoT has entered our personal living spaces, allowing us to use a variety of electronic gadgets at home while improving energy management and enhancing security systems. [13,14]. The IoT has the capability to change various industries, make processes more efficient, and improve the living standards of different communities across the globe [15,16].

**Figure 1:**
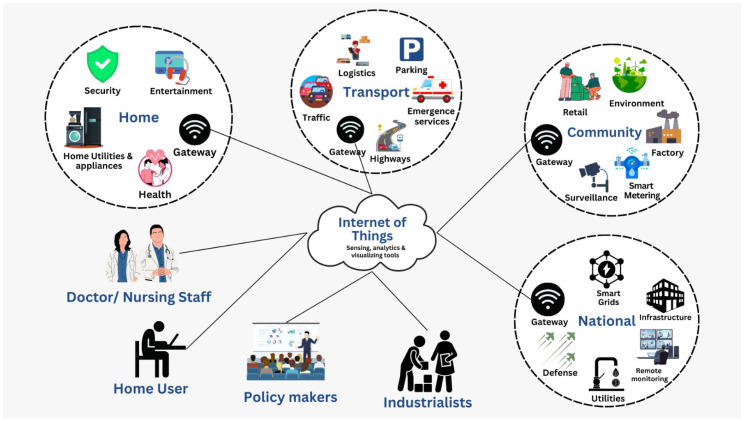
IoT network.

The rapid growth of IoT has also been marked by several challenges, one of the most significant being the issue of cybersecurity [17,18]. People utilize electronics extensively; hence, cyberattacks happen increasingly often. The general purity of IoT systems is in danger because of security loopholes, privacy issues, and attack opportunities [19,20]. As it occurs, the possibility of launching attacks in the RPL is a serious security concern. These assaults may cause significant gaps and illegal access, undermining the stability of data transmission in networks. [21]. The increasing safety issues have been addressed by IoT collaborative learning [22,23,24]. Federated learning is a type of machine learning (ML) that uses individual device training to lower the privacy concerns related to centralised data processing [25,26]. This joint method allows devices to gain knowledge from data without jeopardizing private information, hence boosting security in distributed settings [27,28]. Integrating federated learning into the monitoring method for sending threats in the RPL protocol provides a flexible way to protect IoT networks [29,30,31]. Figure 1 presents the framework of an IoT setup.

### 1.1. Motivation

The accumulation of IoT devices has transformed industries, resulting in substantial security issues. This study aims to address these issues by developing cutting-edge ML, federated learning (FL), and deep learning (DL) models to detect and alleviate security issues. Motivated by the restrictions of existing IoT security techniques, this research emphasizes overpowering issues, including scalability, energy efficiency, and real-time computational abilities. The aim is to project a robust FL model that can handle dynamic IoT settings, guarantee data secrecy, and minimize communication overhead or latency. Moreover, enhancing DL models for resource-inhibited IoT devices without conciliatory performance is critical. By addressing these concerns, this study aims to improve IoT systems’ security, efficacy, and scalability to support state-of-the-art applications and secure against developing cyber threats.

### 1.2. Use Case: Selective Forwarding Attacks in Smart Healthcare Systems

Smart health technologies are important for the enhancement of one’s wellness and personal health [32]. The devices primarily involve in-built moisture sensors for biometric data analysis, which include heartbeat rate, sleeping habits, and activity levels. These types of devices, like remote patient monitoring systems, play a crucial role in enhancing patient results and caring for chronic conditions. Devices of the type described above typically include implanted sensors to monitor blood pressure, glucose levels, and oxygen saturation. For instance, an ECG sensor, used in remote patient monitoring systems for heart problems, may detect heart rate and any anomalies the instant they happen. By checking these crucial signs often, patients and health care providers will be able to anticipate and identify potential health risks early enough to modify treatment plans where necessary and act promptly to avert adverse outcomes. By means of such real-time information, individuals can take a more active role in maintaining their own well-being while doctors obtain suggestions on how best to provide personalized treatments. Nevertheless, the interconnection of smart healthcare systems has resulted in serious cybersecurity risks, which need to be looked into if patient safety and information confidentiality are to be ensured.

When a scenario is considered, there could be a number of security reasons regarding unauthorized access to the IoT devices during a cyber attack. For example, compromising communication systems may involve intentionally altering dosages for prescribed drugs in such a way that it places individuals in harmful situations. Their records can be used for fraud and identity theft or to commit other illegal offenses. Also, patients’ personal information can be accessed with their details, violating their basic rights. Moreover, interruptions to healthcare services induced by cyber-attacks on smart healthcare infrastructure might have far-reaching consequences, jeopardizing patient care delivery and weakening faith in digital healthcare solutions. Therefore, comprehensive cybersecurity measures are necessary to guard against such attacks and preserve the integrity and dependability of smart healthcare devices and health management. The study provides a federated learning-based detection mechanism for fighting selective forwarding attacks in IoT networks to identify and mitigate routing risks in real time, utilizing feature extraction, normalization, and model training using deep learning techniques.

The primary aim of this research is threefold:

1. To perform an extensive review of the literature on selective forwarding attacks and the current strategies for detecting them;
2. To create a simulation environment for assessing the effects of selective forwarding attacks on RPL-based IoT networks, and;
3. To devise a security framework incorporating federated learning to improve detection accuracy of the system.

The paper is organized in different sections as described: Section 2 provides a detailed literature review followed by the research gaps in the existing studies. In Section 3, a detailed view of the proposed framework and methodology is presented. Section 4 details the description of the results of all the learning models and their state-of-the-art comparison and discussion. Finally, in Section 5, a brief conclusion of the study is provided along with future research directions.

## 2. Related Work

A comprehensive analysis of the weaknesses and challenges of the IoT is detailed in this section. It categorizes ML models and investigates the applications of FL and DL in increasing IoT security.

### 2.1. Security Challenges in IoT Attack Mitigation Studies

Table 1 presents a comprehensive analysis of the latest studies about mitigating security threats in the IoT. The study in [33] trained the convolutional neural network (CNN) model ResNet and transformed network traffic data into pictures to identify DoS and DDoS attacks. The work in [34] introduced a radio-based RF jamming mitigation platform that uses programmable beam-steering antennas at the physical layer and carries out real-time jammer categorization. The findings indicated that this proposed study outperformed the current state-of-the-art technique by 9% in binary classification accuracy. Utilising UNSW-NB15 data, the study in [35] investigates flow, MQTT, and TCP feature clusters. This work used supervised ML approaches like RF, SVM, and ANN for the cluster analysis. The outcomes show that, while using RF, the proposed approach achieved an accuracy of 98.67% and 97.37%. A classification accuracy of 96.96%, 91.4%, and 97.54% was obtained through RF for the top features from both clusters, TCP features, and flow & MQTT features, respectively.

Another work in [36], used nine well-known machine learning algorithms to conduct tests on an IoT dataset. Many machine learning approaches were evaluated in this work: RF, LR, DT, KNN, BG, SVM, NB, NN, and ST. The work shows that the proposed method identifies IoT malware with a perfect accuracy of 100% by using DT, SVM, RF, and Bagging classifiers. It achieves an approximately 99.9% accuracy for LR, NB, KNN, and Neural Networks. However, the Simple Tree classifier only achieves an accuracy of 28.16%. The research conducted in [37] employed a structured approach to simulate the entire transmission process and featured a generation technique to generate artificial training data (ATD). Two Support Vector Machine (SVM) classifiers were assessed: a conventional binary SVM classifier (TC-SVM) and a single-class SVM classifier (SC-SVM). The results suggest that the accurate calibration of parameters is essential to obtain a detection probability of 95% for those attempting to listen in on private conversations.

### 2.2. Federated Learning Use Cases in IoT

The provided table, labeled as Table 2, classifies federated learning models that aim to enhance the security of IoT devices. The work conducted in [38] presents an efficient federated defense approach known as FDA3. This study was constrained by the communication delay. The paper [39] introduced DÏoT, an autonomous and self-learning distributed system that employs federated learning to detect anomalous intrusions in IoT devices. DÏoT achieved a remarkable 95.6% rate of identifying devices infected with the Mirai virus in just 257 ms. The study conducted in [40] employed a ML-based IDS implemented within the framework of the IoT industry. A constraint of the study was the absence of exploration into scalability concerns. The study in [41] demonstrated the earliest research on intrusion detection in a simulated setting. This study has shown that the federated technique is highly effective in classification accuracy, computation, and communication cost when applied to the AWID intrusion detection dataset. The major downside of this methodology is the need for empirical performance analysis. The study conducted in [42] presents MV-FLID, a FL approach for intrusion detection in IoT networks. This paper does not include a validation of the performance of FL in comparison to the ML and DL approaches. The research done in [41] employed LiM, a privacy-centric system for categorizing malware that relies on federated learning. The cloud server achieved an excellent F1 score of 95%, indicating high accuracy. However, the research needed to incorporate the convergence of the learning process, which is a drawback of the study.

The authors in [43] evaluate the efficacy of a FL-based IDS that uses a multiclass classifier to detect various forms of assaults in IoT environments with diverse data distributions. This highlights the challenge of connecting IoT devices, indicating the need for further investigation and research. The study in [44] developed a resilient framework for securely transferring data among IoT devices. The SecureIIoT model exhibited exceptional precision, with accuracy of 99.79% when binary classification was used to identify attacks. Nevertheless, its drawback lies in the requirement for testing on larger datasets. The study in [45] proposed a technique that utilizes federated learning to detect IoT devices affected by malware. This approach involves the utilization of the N-BaIoT dataset. This technique needs to address the topics of energy efficiency and learning capacity. The study in [46] presented an FL model integrating a dual-reputation reverse auction to improve security and select edge nodes in IoT settings. Their method employs a reputation-bid ratio-based algorithm for edge node selection, a flexible dropout accumulation technique to prevent malicious attacks, and blockchain for repute administration. Simulation outcomes imply that their model outperforms standard models in accuracy and reaction to assaults.

### 2.3. Deep Learning Models for IoT Applications

Table 3 presents a comprehensive analysis of the deep learning models’ effectiveness in various IoT applications. The study in [47] employed a TensorFlow deep neural network to identify pirated software by detecting instances of source code copying. According to the findings, the suggested model achieved a classification accuracy of 97.46%. The study in [48] presented a deep learning framework developed explicitly for an IoT-centered infrastructure in a secure smart city. Blockchain technology is employed to establish a decentralized environment in the communication phase of Cyber-Physical Systems (CPSs). The results showed a precision value of 0.7244, a recall value of 0.7078, and an F1 score of 0.7118, indicating a high level of scalability. The study in [49] employed a FFDNN Wireless IDS equipped with a WFEU. Using the UNSW-NB15 dataset, the authors achieved an accuracy of 87.10% for binary classification and 77.16% for multiclass classification. The AWID dataset demonstrated an overall accuracy of 99.66% for binary classification and 99.77% for multiclass classification. Another study in [50] presents a sequential methodology that collects network layer attributes through TCP dump packets and application layer attributes through system functions. The study employed Text-CNN and GRU methodologies to derive supplementary characteristics from the data. The statistics indicate that the model achieved an F1 score of 0.98%. The research in [51] devised a novel intrusion detection system for IoT networks, employing profound learning principles to categorize data flow accurately. The model is designed for both multiclass as well as binary classification. The study achieved a prediction accuracy of 99.5% for the NSL-KSS dataset, 99.3% for the CIDDS-001 dataset, and 99.1% for the UNSWNB15 dataset. The research in [52] presented an IoT-IDCS-CNN that harnesses the functionalities of convolutional neural networks. The results showed a classification accuracy of 99.30%, as well as multiclass classification accuracy of 98.20%. The system underwent verification by K-fold cross-validation and evaluation, utilizing parameters from the confusion matrix.

### 2.4. Research Gaps

There are several security frameworks available in the existing literature; however, it is essential to evaluate some critical concerns carefully. That includes the obstacles in FL-IoT that arise from the convergence issues in integrating learning and communication. These issues are created by the IoT network’s sensitivity to variations in sensing environments. The limitations of IoT in terms of storage and computational capability lead to longer delays and fluctuating synchronization. Current data transport techniques frequently increase energy usage, particularly when utilizing GPUs, diminishing overall energy efficiency. The trade-offs include the careful consideration of several factors, including safety, energy efficiency, cost-effectiveness, and accessibility, while also prioritizing security. It can be challenging, since existing models may prioritize security above specific metrics. Moreover, the integration of privacy-conserving techniques in FL is still in its initial stages, with inadequate study into corresponding data privacy and model efficiency. An additional area necessitating further study is the energy efficacy of DL models, as the high computational costs of the models can lead to substantial energy consumption, leading to challenges for battery-functioned IoT devices. Additionally, the compliance of security models to emerging threat/vulnerability landscapes and background responsiveness in IDSs is vital for developing robust IoT security frameworks. Ensuring equilibrium among these parameters is crucial to enhance security without sacrificing other essential features.

## 3. Research Methodology

**Figure 2:**
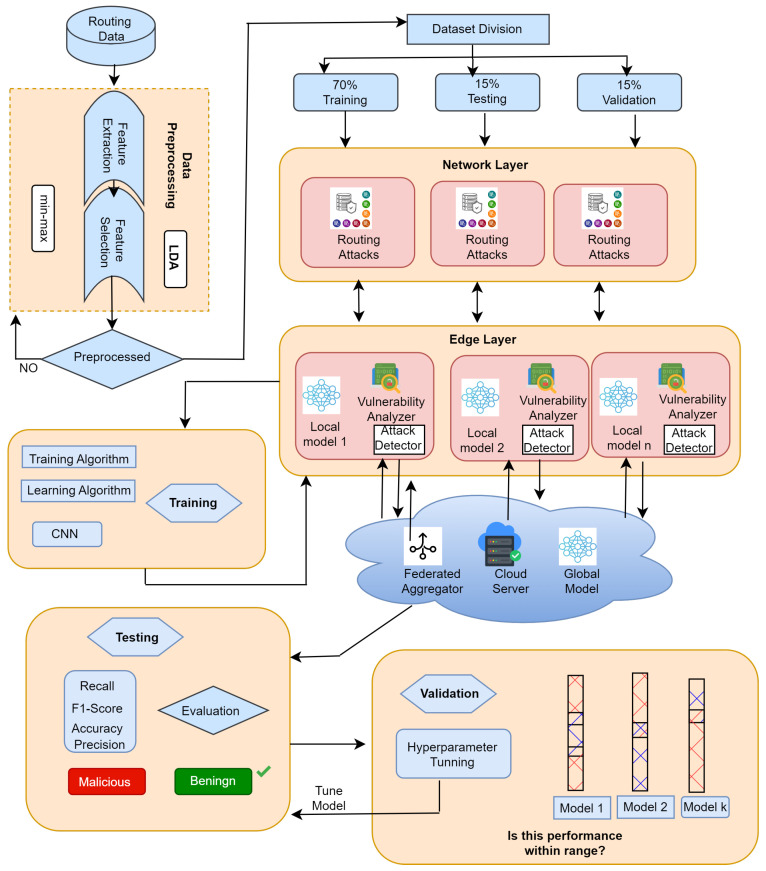
Research framework.

In this section, a comprehensive description of the research methodology is presented. Figure 2 provides a graphic representation of the whole research framework and summarizes the workflow and interconnected elements of the federated learning model that was put into practice.

### 3.1. Dataset Description and Pre-Processing

A detailed description and pre-processing steps of the dataset are mentioned in this section.

**Figure 3:**
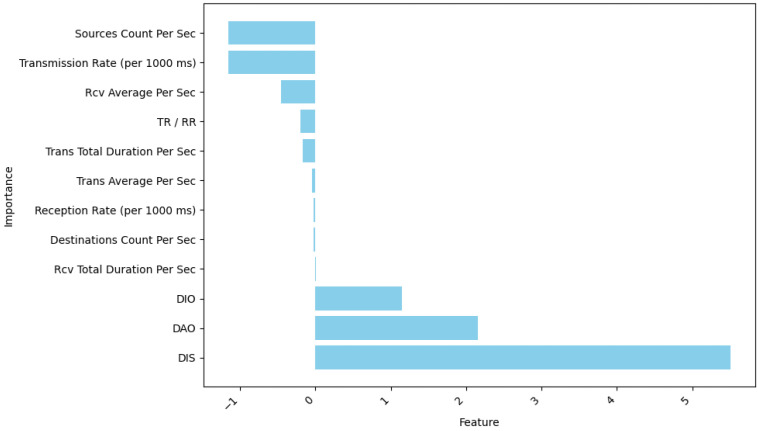
Feature importance from Linear Discriminant Analysis (LDA).

1. Dataset Description: This study utilizes a publicly accessible dataset called IoT Routing Attack Dataset (IRAD) [53]. The dataset comprises authentic data, containing both harmless and the most current widespread attacks. The table provides a comprehensive account of the quantity and dimensions of each occurrence; see Table 4.
  In this study, three samples of dataset are used, each including two distinct classes: the malicious and benign samples.
2. Dataset Split: Training, Validation, and Test: During the data sampling step, a meticulously chosen subset of 150,000 samples has been gathered from the dataset. The provided subset has been methodically partitioned into three sets to improve the reliability of training, testing, and validating the model. More precisely, 70% of the samples have been allocated to the training set, which forms the basis for the model’s learning process. Afterwards, 10% of the samples were assigned to the validation set, which is crucial as it helps in tuning up the model’s performance. This test set contains the leftover 20% of the data; it checks how well our model can be applied outside the existing data. With this stratified partitioning strategy, there is no bias or favoritism in any of the training set, validation set or test sets used for model assessment. See Table 5.
3. Dataset Pre-processing: A wireshark analyzer was used to convert the packet capture (PCAP) files to a comma-separated value (CSV) format. A preprocessing script has been developed, as will be described.
  - Feature Extraction and Selection: This research focuses on the process, which is thorough in regards to obtaining selections and developing features, struggling heavily to refine dataset features and aiming at overcoming the associated danger of overfitting. The use of Linear Discriminant Analysis (LDA) enhanced the separability of different classes by representing each subset with a single feature, allowing for the distinction between attack and benign classes, as demonstrated in Figure 3. By intentionally excluding identifiers, bias was minimized, thereby assuring a thorough and unbiased investigation. The IRAD datasets, which include qualitative and quantitative features, were carefully converted to be compatible with numerical learning methods. The significant analysis and adjustment of DAO, DIO, and 6LoWPAN characteristics highlight their crucial functions in the Routing Protocol for RPL. The combination of harmless and destructive datasets maintained consistency in structural characteristics, and using Scikit-learn, properties showing strong correlations to the output variable were specifically separated. This comprehensive approach emphasizes the thorough identification and selection of characteristics to achieve research goals within the specific field. Three attacks were evaluated, focusing on the reception elements and transmission of 6LoWPAN protocol packets and DIO control messages. The selection of relevant attributes was carried out using deep neural networks, Pearson correlation coefficients, and histograms. The DNN technique was used to evaluate the value of the recovered characteristics and identify the most effective number of neurons. The information on the features is provided in Table 6.
    (1) $$S=\frac{p(\Sigma ab)-(\Sigma a)(\Sigma b)}{\sqrt{\left[p\Sigma c^{2}-{\left(\Sigma c\right)}^{2}\right]\left[p\Sigma d^{2}-{\left(\Sigma d\right)}^{2}\right]}}$$
  - Feature Normalization: The feature normalization procedure is used to standardize datasets and populate them into an even range. The min–max scaling procedures were the most direct, uncomplicated, and adaptable method for normalizing the values of the selected attributes. The newly introduced feature, designated as Y*, is adjusted to span a range of values between 0 and 1. RPL stands for the values that have been normalized. The variable “RPL.FACTOR” denotes the starting value of the feature, whereas “RPL.FACTORmin” and “RPL.FACTORmax” indicate the minimum and maximum values of the selected features, respectively, as shown in Equation (2)
    (2) $$Y^{*}=\frac{\mathrm{RPL}.\mathrm{FACTOR}-\mathrm{RPL}.\mathrm{FACTORmin}}{\mathrm{RPL}.\mathrm{FACTORmax}-\mathrm{RPL}.\mathrm{FACTORmin}}$$
    By using bagging to combine unbiased variables, a model with decreased variance was attained. A classifier, which is a feed-forward artificial neural network, was used to examine specific properties. The network consisted of at least three nodes in each layer and utilized various activation functions.

### 3.2. Local Models’ Training

In this study, each IoT device goes through a systematic and personalized training program after careful system setup and thorough device selection. The server employs strategic configuration and initialization during the local training and update stage to introduce a new model. Subsequently, this model is distributed to individual IoT users, commencing a decentralized training procedure. Users, separate entities inside the IoT network, independently improve their local models by utilizing unique datasets. This reiterative process includes several rounds of updates, during which every device improves its local models based on unseen data and the response received from the federated global model. This involves updating the training data to minimize the loss function *F*, as specified by the optimization equation:

(3) $$W_{p}^{*}={\mathrm{argmin}}_{w_{p}\in P}F,\;p\in P$$

In this context, $W_{p}^{*}$ represents the optimal model parameters for a particular user *p* in the federated learning (FL) process. The use of localized training ensures that every IoT device, operating as an autonomous agent, may autonomously enhance its model. Consequently, the enhanced individual models together enhance the intelligence of the aggregate global model. Methods including differential privacy and secure cooperative computation are used to ensure validity. Stringent measures are used during this process to ensure the utmost privacy and integrity of the data. Once models have been trained, they are uploaded and methodically merged at the base station, which is a crucial step in federated learning in the IoT context. The models undergo training utilizing five deep learning approaches to improve analysis and simplify result comparison.

1. Logistic regression (LR) The statistical deep learning method is utilized for binary classification to predict the likelihood of a routing assault using the IoT Routing Attack Dataset (IRAD). The logistic regression model is formally defined as follows:
  (4) $$P\left(y=1|X\right)=\frac{1}{1+e^{-(\beta_{0}+\beta_{1}X_{1}+\beta_{2}X_{2}+\ldots +\beta_{n}X_{n})}}$$
2. K-nearest Neighbors (KNN) The KNN approach is a classification strategy that assigns a class to a data point based on the majority class of its nearest neighbor in the space of the feature. This approach is agnostic to any specific assumptions about the data distribution. The projected class for a certain data point, X, is mathematically defined by the class that appears most frequently among its k-nearest neighbors.
3. Support Vector Machine (SVM) SVM is an ML classification and regression analysis technique. It belongs to the supervised learning domain, which implies that it necessitates labeled data for model training. The objective is to ascertain a hyperplane that effectively partitions the data into definite classes. The SVM technique aims to mathematically identify the hyperplane (H) with the largest margin that can accurately divide the classes.
4. Naıve Bayes (NB) The NB method is a probabilistic approach that applies Bayes’ theorem and assumes attribute independence. It is commonly used for activities that need categorization. The Naive Bayes model is formally defined as follows:
  (5) $$P\left(C_{k}|x\right)=\frac{P\left(x|C_{k}\right)\cdot P\left(C_{k}\right)}{P(x)}$$

### 3.3. Global Model Aggregation

Following the use of DNN approaches, several local models were trained. The weights of these models were then aggregated and consolidated worldwide using the Federated Averaging method. It is a collaborative method for merging model weights without directly accessing specific variables. The system employs federated learning, storing individual models on several devices. The training process is distributed, indicating that several servers are utilized. The local models that have undergone training possess weights. Federated Averaging has been employed to merge the data, resulting in a more robust and widely applicable global model.

1. Federated Averaging: following the individual clients’ training and updating of the model, the server combines the models and calculates the new global model using Equation below:
  (6) $$\mathrm{global}\;\mathrm{model}\leftarrow \sum_{i=1}^{N}\left(\frac{N_{i}}{N}\times \mathrm{local}\;{\mathrm{model}}_{i}\right)$$
  This technique ensures that the global model mirrors the cooperative developments from all local models, upholding high efficiency while conserving data secrecy.
  In addition, the loss function can be reduced by handling the optimisation complexity given in the Equation below:
  (7) $$p=\min_{w_{p}\in P}\frac{1}{p}\sum_{p=1}^{p}w_{p}^{\prime }$$

The aggregated global model influences the distributed character of IoT devices, compounding their insights without conciliating the distinct data secrecy of every device.

### 3.4. The Cross-Validation of the Models

Cross-validation is used to validate the model’s results by splitting the dataset into *k* subgroups, or “folds”. In this research, *k*-fold cross-validation was used, where every fold is successively employed as a validation set, though the other k−1 folds are employed for training. This procedure is repeated *k* times, and the middling performance metrics are calculated to measure the model’s applicability. The average efficiency metric *M* is considered as follows:

$$M=\frac{1}{k}\sum_{i=1}^{k}M_{i}$$

where $M_{i}$ is the metric for the *i*^th^ fold. This method makes sure that the model’s efficiency is assessed broadly for other subsections of the dataset, thus delivering a dependable approximation of its efficiency.

### 3.5. Evaluation Parameters for Methodology

An all-inclusive set of metrics is used to evaluate the tested technique regarding its resistance and how well it works:

- Accuracy: The accuracy is a key factor used to measure to what extent the model’s predictions are correct, providing a strategy to analyze its overall performance.
- Precision: This statistic examines the precision of positive predictions, emphasizing the model’s ability to reduce false positives. It is especially pertinent in situations when accuracy is a crucial factor. This parameter checks the precision of positive predictions in reducing false alerts; it is important especially when accuracy matters most.
- Recall: Recall reveals how many instances are correctly identified versus misclassified data points, taking into consideration sensitive issues so that the system responds to relevant cases as expected without classifying anything incorrectly, and without caring about sensitivity and specificity. In preventing further occurrences of error type II, this value is required.
- F1 Score: F1 score provides the mean of both accuracy and recall, offering a comprehensive evaluation of the FL models. This is obtained through taking both false positives and false negatives into consideration, hence giving a balanced view.
- Confusion Matrix: A visual portrayal emphasizes the allocation of true positive, true negative, inaccurate positive, and erroneous negative predictions, offering a full overview of the model’s efficacy across multiple categories.

These well-selected evaluation criteria, taken together, provide a detailed and complex picture of the performance of the approach in many aspects, guaranteeing a thorough evaluation of its effectiveness.

### 3.6. Federated Learning Algorithm

The research methodology used in this work is described in Algorithm 1. Local model training is performed at the Fog layer and the CPS layer, two separate levels of the system. Key parameters like the number of communication rounds, local models, learning rate, and aggregation technique are set during the initialization phase. Each CPS node defines its local datasets, and a global model is initialized. The Fog layer functions throughout multiple runs. The local models obtained from CPS nodes are combined to update the global model. It is subsequently broadcast to all nodes and transmitted separately to each CPS node. The local model training CPS layer specifically targets individual CPS nodes. Every node starts by initializing a local model and then updates it during communication rounds using the global model and its local dataset. The optimized local model is, after that, returned to the Fog layer. This method facilitates collaborative learning while ensuring privacy, as the global model is updated by aggregating information from all CPS nodes. The organized exchange of information between the Fog and CPS layers enables the implementation of federated learning inside the CPS framework.

### 3.7. Implementation Details

This section presents the computational settings for implementing and evaluating the proposed model. The implementation used Google Colab to produce and train an FL model using numerous tools and frameworks. The implementational configuration in the Google Colab (USA) setting used the workings of the subsequent hardware systems: Google Colab’s RAM capability differs depending on the selected runtime nature, ranging from 12 GB to 25.5 GB. The CPU resources have various cores, enabling simultaneous model implication and training processing. The most appropriate GPUs for DL workloads are NVIDIA Tesla T4 (USA). The subsequent hardware requirements were essential to implement FL in the Google Colab setting efficiently, using the TensorFlow Federated (TFF) framework, TensorFlow (tf. keras) or PyTorch libraries, and NumPy. Consuming RAM, CPU, GPU, and disc resources enabled the effective and swift training of models, allowing for the effective building and analysis of the FL model through the experiment. Details are mentioned in Table 7.
**Algorithm 1** Federated learning with Selective Forwarding Attack Detection. 1:**Input:** Local datasets $\left\{D_{1},D_{2},\ldots ,D_{n}\right\}$ for each CPS node 2:**Output:** Global Model $M_{\mathrm{global}}$ 3:**Initialization:** 4:Set communication rounds *T*, local models *M*, learning rate η, aggregation method ϕ 5:Initialize global model $M_{\mathrm{global}}^{\left(0\right)}$ 6:**Fog Layer Processing:** 7:**for** t=1 to *T* **do** 8:   Receive local models from CPS nodes: $\left\{M_{1}^{\left(t\right)},M_{2}^{\left(t\right)},\ldots ,M_{n}^{\left(t\right)}\right\}$ 9:   Aggregate models: $M_{\mathrm{global}}^{\left(t\right)}=\phi \left(\left\{M_{1}^{\left(t\right)},M_{2}^{\left(t\right)},\ldots ,M_{n}^{\left(t\right)}\right\}\right)$10:   Broadcast global model $M_{\mathrm{global}}^{\left(t\right)}$ to all CPS nodes11:**end for**12:**CPS Layer for Local Model Training:**13:**for** i=1 to *n* **do**14:   Initialize local model $M_{i}^{\left(0\right)}$15:   **for** t=1 to *T* **do**16:     Receive global model $M_{\mathrm{global}}^{\left(t\right)}$17:     Train local model $M_{i}^{\left(t\right)}=\mathrm{Train}\left(M_{i}^{\left(t-1\right)},D_{i}\right)$ using KNN, LR, SVM, NB18:     Transmit local model $M_{i}^{\left(t\right)}$ to Fog layer19:   **end for**20:**end for**21:**Final Aggregation:**22:Finalize the global model $M_{\mathrm{global}}$

## 4. Results

This section contains the analysis and results found using the method and gives a detailed look at the outcomes achieved as a consequence of its application.

### 4.1. KNN

This section constitutes the significance and the impact of the KNN joined with its usability. In addition to that, it contains the particular performance figures of the same technique in the deployment framework.

**Figure 4:**
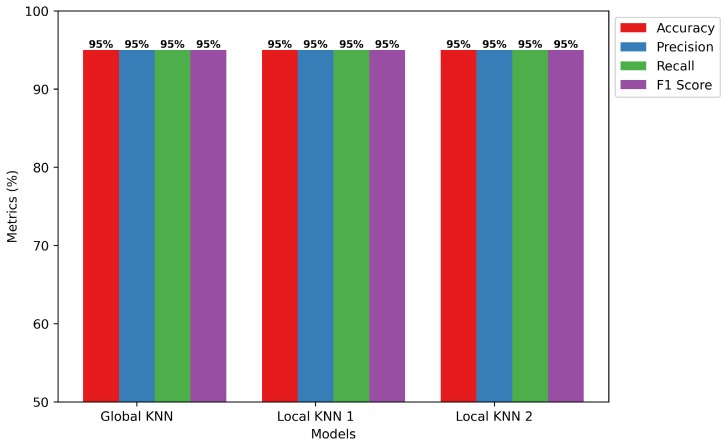
KNN training model.

**Figure 5:**
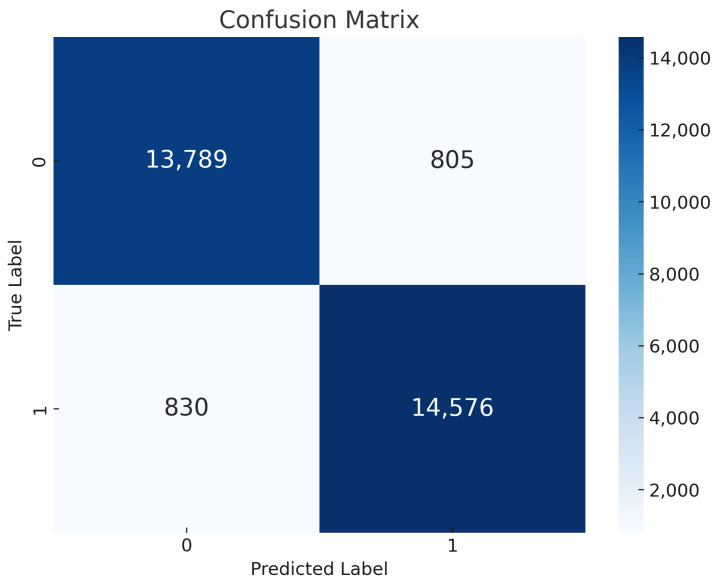
KNN confusion matrix.

1. Global vs. Local Model Training: The outcomes of our study are shown in Figure 4. In several assessment categories, they demonstrate noteworthy accomplishments. The local and global models scored an amazing 95% for accuracy, recall, precision, and F1 score. The local models are made especially to handle the complexity of their particular IoT environments, effectively differentiating between normal network behavior and potentially dangerous behavior. Smooth data integration from several local models by the global model enhances its understanding of the dynamics of IoT networks. The global model replicates the outstanding performance of the local models through the use of this cooperative technique, producing an amazing 95% in all assessment criteria. Considering their overall amazing accuracy, recall, precision, and F1 score measures, the models are trustworthy in handling complexity in the dataset.
2. Confusion Matrix of KNN: Figure 5 displays the efficacy of the KNN model. Based on the data, the model accurately predicted 13,789 instances out of 28,000 as “True 0”, which signals regular network functioning. In addition, the program properly identified 14,576 incidents as “True 1”, indicating incidences of selective forwarding attacks. Yet, it yielded 805 misclassifications, marked as “predicted 0” that were actually “true 1”; additionally, 830 identifications were wrongly flagged as “predicted 1” when in reality, they were “true 0”. However, there are infrequent misclassifications, but it carries out very well on the whole by guessing right in many situations and making only a few false ones. Its competence and potential in enhancing security against malicious attacks in IoT networks is highlighted by the impressive performance of the KNN model on recognizing selective forwarding attacks instances on IRAD dataset.

### 4.2. Logistic Regression (LR)

This section summarizes the findings relevant to the Logistic Regression (LR) algorithm, explaining the individual contributions and their influence on the overall outcomes of the federated learning architecture that was put into place.

**Figure 6:**
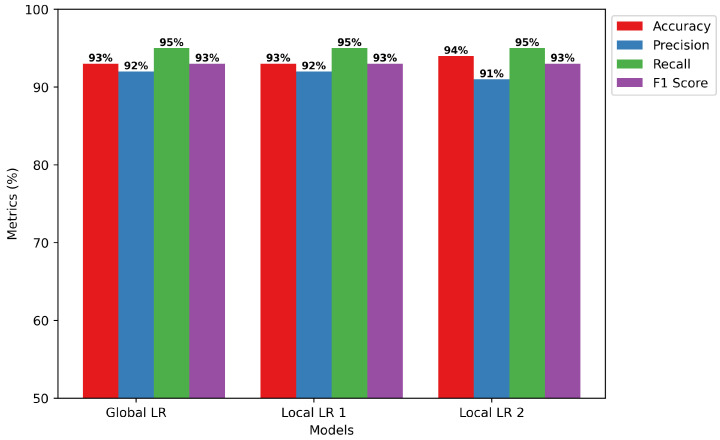
LR training model.

**Figure 7:**
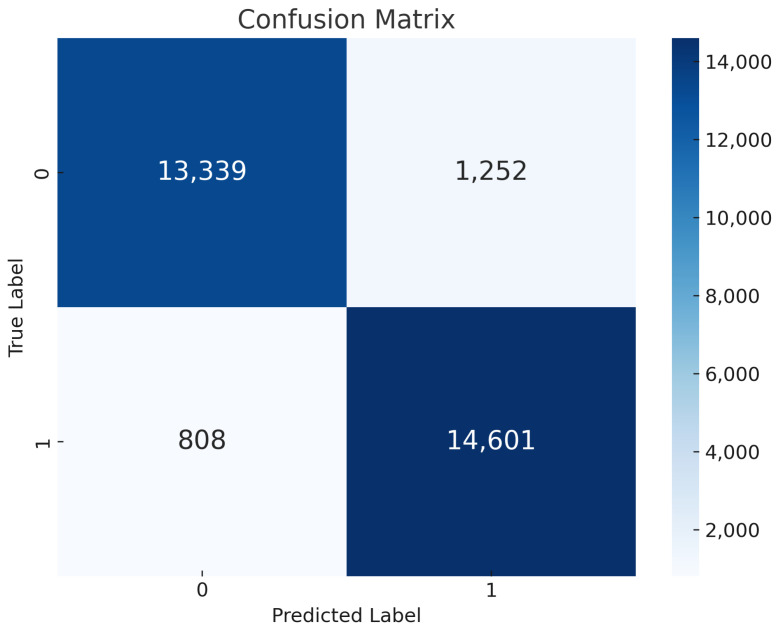
LR Confusion Matrix.

1. Global vs. Local Model Training: The remarkable global and local performance of the logistic regression model is shown in Figure 6, which also shows its usefulness in binary classification problems. The model shows remarkable accuracy rate of 93% with precision, recall, and F1 score values of 92%, 95%, and 93%, in that order, globally. This emphasizes its ability to classify events and effectively document accurate, positive outcomes. At the local level, both local models consistently achieve high levels of accuracy, precision, recall, and F1 score. Some measures show tiny gains. Specifically, Local LR 2 is notable for its increased accuracy of 94%, while it has a slightly lower precision of 91%. However, the global logistic regression model performs better than both local models when evaluating the combined results, confirming its superiority across all measures. By adopting an integrated perspective, the global model emerges as the most advantageous option, capitalizing on the advantages of local variations to obtain superior overall performance in binary classification tasks.
2. Confusion Matrix of LR: The confusion matrix displayed in Figure 7 provides a detailed assessment of the effectiveness of the logistic regression model. The model has impressive accuracy in its predictions, as seen by the significant number of occurrences accurately identified as either absence (13,339 instances) or presence (14,601 instances) of selective forwarding assaults, indicated by true labels 0 and 1, respectively. In addition, the model has a significantly low misclassification rate, with just 1252 examples improperly classified as not having selective forwarding attacks and 808 occurrences mistakenly labelled as having such attacks. The little difference between the predicted and real labels highlights the model’s strong performance and ability to accurately distinguish between normal network behaviour and malicious selective forwarding actions. Strong sensitivity and specificity—qualities essential for preserving the integrity and security of IoT installations depending on the RPL protocol—are shown by the model’s precision. Thus, our results provide a solid foundation for the use of FL-based detection systems, a potent technique of fortifying IoT networks against the dangerous threat of selective forwarding attacks.

### 4.3. Support Vector Machine (SVM)

This section briefly describes the outcomes of the SVM algorithm, emphasizing its purpose and impact on the implemented federated learning architecture.

**Figure 8:**
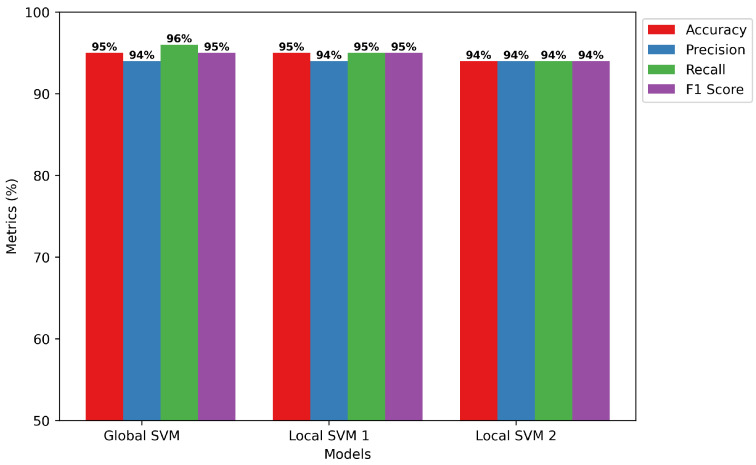
SVM Training Model.

**Figure 9:**
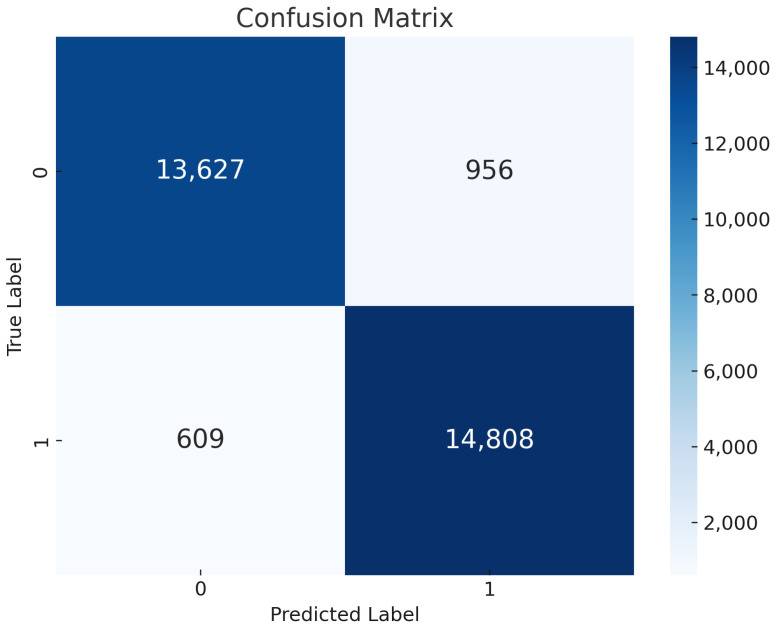
SVM Confusion Matrix.

1. Global vs. Local Model Training: The SVM model’s evaluation parameters provide outstanding performance on a number of measures, as Figure 8 illustrates. The model predicts with a high overall accuracy level of 95%. Besides, the model demonstrates its ability to provide accurate and trustworthy positive identifications with an accuracy rate of 94%. At the same time, a 96% recall rate implies that it recognizes selective forwarding attacks taken as prominent instances. The 95% F1 score, for false negatives and false positives, shows that generally the model has no problem balancing between precision and recall. Taken together, it is evident that SVM can detect patterns that indicate selective forwarding attacks.
2. Confusion Matrix of SVM: The confusion matrix provided in Figure 9 illustrates the high performance of the SVM model while applying federated learning for recognizing selective forwarding attacks in IoT networks with the RPL protocol. The program displays exceptional accuracy in accurately recognizing scenarios that do not include selective forwarding attacks, totaling 13,627 occurrences. Furthermore, it is quite accurate in detecting the occurrence of these assaults, identifying 14,808 instances as selective forwarding behavior. The results demonstrate the model’s exceptional sensitivity and specificity, as well as its ability to successfully strengthen IoT networks against hostile behavior. The ability to accurately distinguish between safe and malicious network data is critical for improving the security of RPL-enabled IoT setups. This provides a solid foundation for future optimization efforts to eliminate misidentifications and improve detection systems.

### 4.4. Naive Bayes (NB)

In this section you will find short descriptions that will tell you what the Naive Bayes (NB) algorithm’s results are, so as to underline its value in the context of federated study.

**Figure 10:**
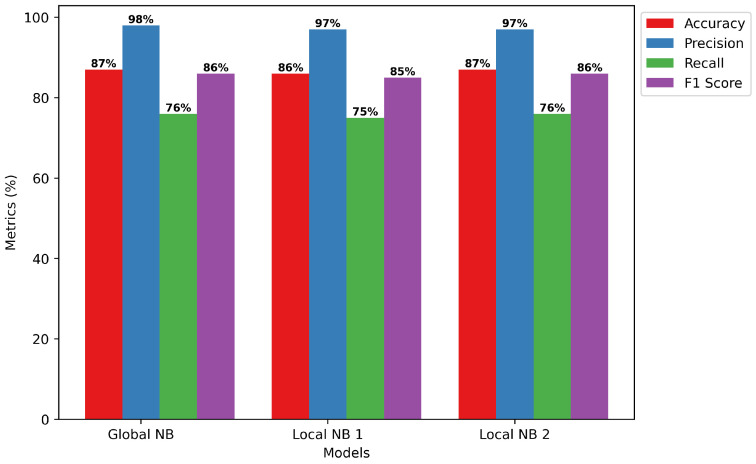
NB training model.

**Figure 11:**
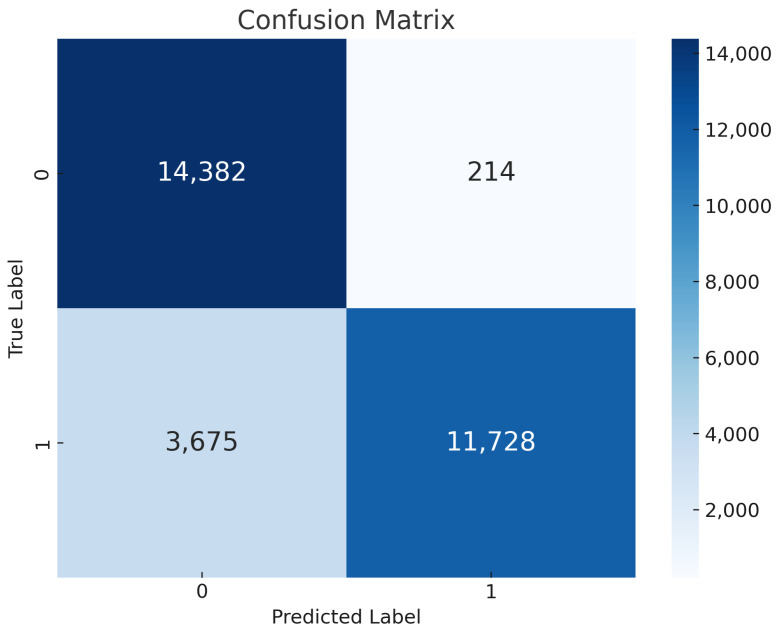
NB confusion matrix.

1. Global vs. Local Model Training: In many aspects, the Naive Bayes classifier performs admirably according to the assessment criteria shown in Figure 10. The model suggests a considerable degree of general correctness in its predictions, with an accuracy of 87%, or the percentage of properly identified instances. For global and local cases, respectively, the model shows that it can provide accurate affirmative identifications with remarkable precision scores of 98% and 97%. Furthermore, the model demonstrates competence in detecting pertinent cases of selective forwarding attacks, even though it has much lower recall scores of 76% and 75% for the global and local examples, respectively. Computed as the mean of accuracy and recall, the F1 scores always reach 86%. This suggests a performance in controlling the trade-off between false negative and false positive that is balanced. The results show that the Naive Bayes classifier is quite good at spotting trends that point to selective forwarding attacks in RPL-controlled IoT networks. This highlights the promise of the classifier as a trustworthy detection technique in the particular research field.
2. Confusion Matrix of NB: We use the confusion matrix presented in Figure 11 to extensively assess the efficacy of a Naive Bayes classifier in identifying selective forwarding attacks in RPL-enabled IoT networks. Although the model misclassifies 14,382 examples as such, it is remarkably accurate at correctly detecting events without sending out assaults only in certain cases. Furthermore, the detection of selective forwarding attacks is quite sensitive; 11,728 examples of this behavior have been found. It is shown that, save from a few instances of false positives (214) and false negatives (3675) results, the model can usually differentiate between beneficial and detrimental network activity. The results indicate that the Naive Bayes classifier proves to be a trustworthy detection technique to enhance the security of IoT systems with RPL capability. Its ability to thwart selective forwarding attacks has to be enhanced, and misclassifications have to be reduced.

### 4.5. Global Model Performance on Test Data

**Figure 12:**
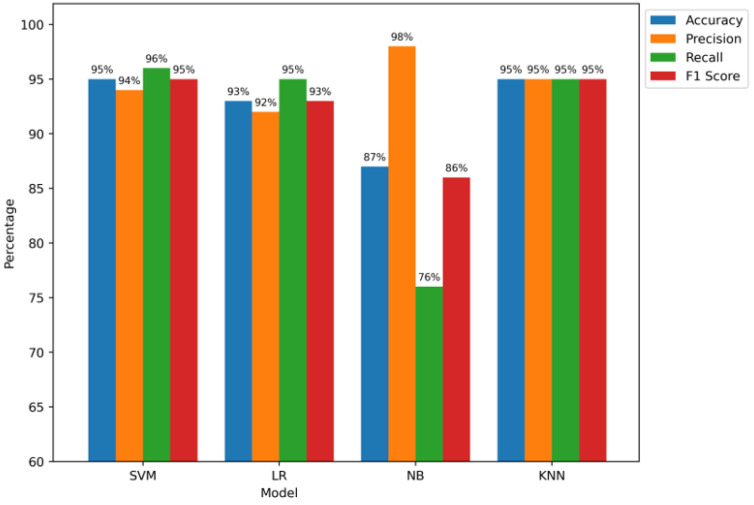
Global models’ performance on test data.

The test dataset is presented in Figure 12 with the evaluation results of four machine learning models: Naive Bayes (NB), Logistic Regression (LR), Support Vector Machine (SVM), and K-Nearest Neighbours (KNN). These findings underline, according to a number of standards, the special performance qualities of every model. The impressive accuracy scores of 95% of the KNN and SVM models, especially, show how well they can categorize situations. The SVM model has an accuracy of 94%; thus, it can accurately identify positive scenarios. Additionally, the stunning 96% recall rate is evidence of how well it captures relevant examples of selective forwarding attacks. What the KNN model shows by reaching a balanced precision, F1 score, and recall of 95% is that it has high classifying accuracy, implying the detection of patterns that possibly indicate selective forwarding attacks. Despite its somewhat lower accuracy of 93%, the LR model shows good precision, recall, and F1 scores of 92%, 95%, and 93%, respectively. This underlines its capacity to accurately identify positive situations and document noteworthy examples of selective forwarding assaults. On the other hand, while attaining a very high precision of 98%, the accuracy of the NB model is noticeably lower, at 87%. This is why the model reduces false positives really effectively. Still, its 86% F1 score and significantly lower recall rate of 76% suggest that it might be difficult to detect every instance of selective forwarding assaults. We finally show how successfully a number of algorithms detect selective forwarding attacks in RPL-based IoT networks. Their limits and benefits are clarified in a significant way.

### 4.6. Global Models’ Performance on Validation Data

**Figure 13:**
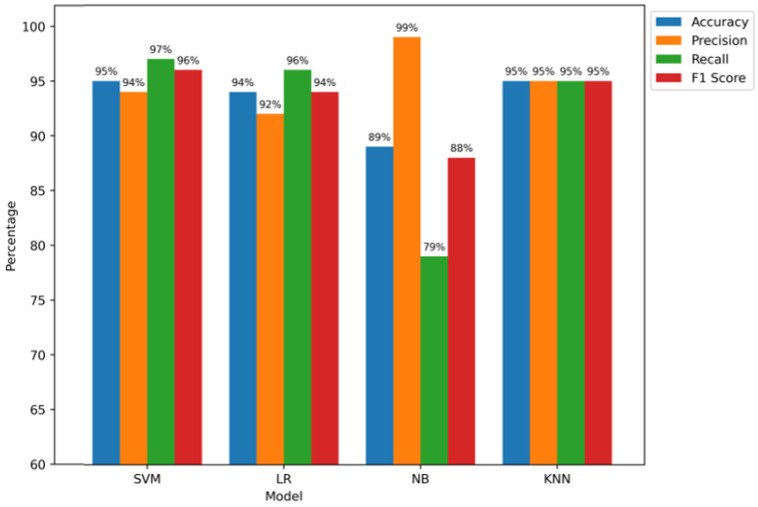
Global models’ performance on validation data.

Figure 13 displays the validation data evaluation results and highlights the differences in performance across several parameters. The remarkable accuracy rate of 95% of the SVM and KNN models, especially, highlights their ability to categorize traffic accurately. With a precision of 94%, the SVM model shows that it is quite accurate at detecting affirmative cases. It demonstrates that it can capture significant instances of selective forwarding attacks with a 97% recall rate. With 95% F1 scores, precision, and recall, the KNN model performs in a well-balanced manner. This demonstrates its continuing accuracy in data categorization and usefulness in identifying patterns, suggesting focused forwarding attacks. Though it lags in accuracy at 94%, the LR model shows important precision, recall, and F1 score at 92%, 96%, and 94%, in that order. This shows that it can detect positive cases and record pertinent instances of selective forwarding attacks. In contrast, the accuracy of the NB model differs significantly, at 89%. However, its remarkably high recall at 99% raises the possibility that the system can successfully rule out false positives. Even then, the somewhat lower recall of 79% and F1 score of 88% suggest possible difficulties in accurately recognizing all relevant cases of selective forwarding attacks. These data provide a detailed overview of the models’ performance environment and perceptive details on their applicability and potential for improvement in detecting selective forwarding attacks in RPL-based IoT networks.

### 4.7. State of the Art Comparison and Discussion

The major advantages of the proposed methodology and the current machine learning methods are shown in Table 8. This work presents a novel method with remarkable benefits, such as a decentralized system, enhanced data privacy, and large-scale deployment simplicity. Exceptional performance measurements enhance these qualities in several aspects. The study suggests that SVM and KNN, when employed under FL, attained an amazing 95% accuracy, surpassing the 94% of Logistic Regression, while Naive Bayes (NB) reached only 89%. Furthermore, precision measurements show that at 99%, NB outperforms SVM and KNN at 94% and LR at 92%. Though recall rates vary somewhat, SVM outperforms LR and KNN at rates of 96% and NB at 79%. All models, though, routinely perform quite well when considering the F1 score. In such sequences, KNN scores 95%, LR scores 94%, and SVM scores 96%. The difference shows how considerably FL enhances the capacity of intrusion detection in IoT systems, signaling a new era of reliable cybersecurity solutions that prioritize data privacy, distributed processing, and flexible deployment. Promising results have been obtained from the suggested model applied to machine learning models. SVM and KNN, particularly, reach high accuracy rates of 95% on both the test and validation datasets, with the global models continually showing excellent performance in classification correctness. Furthermore, an analysis of the state of the art emphasizes FL’s advantages over traditional machine-learning techniques regarding data privacy, decentralized processing, and scalability. These results highlight how much FL enhances the security of IoT deployments, particularly by lessening the negative effects of selective forwarding attacks in networks with RPL enabled.

Hyperparameters, including batch size, learning rate, and dropout rate, impacted the performance of the FL-DSFA. The research utilized a learning rate of 0.001, a batch size of 32, and a dropout rate of 0.25 to avoid overfitting and improve model generality. These hyperparameters have a straight-wedged model’s accuracy and convergence pace. The LR of 0.001 ensured steady convergence, though the dropout rate of 0.25 enhanced generalization, despite producing underfitting. The optimization of these hyperparameters was vital for attaining optimal efficacy and performance in the proposed model.

### 4.8. Potential Real-World Applications of FL-DSFA

FL-DSFA proposes a vigorous solution for improving the security of IoT nets for a wide range of cyber threats. In smart homes and cities, FL-DSFA can protect interrelated devices from DDoS assaults by detecting abnormal traffic patterns without risking user data secrecy. In healthcare, this system can secure critical medical devices and patient data from malicious and forbidden access, ensuring the integrity and confidentiality of crucial health data. Furthermore, FL-DSFA can strengthen industrial systems against APTs in industrial IoT settings by unceasingly apprising and refining security models founded on real-time datasets from varied edge/endpoint devices. By exploiting distributed learning, FL-DSFA can offer compliant and robust security procedures personalized to the explicit requirements of several IoT settings, thus improving IoT networks’ inclusive robustness and dependability.

## 5. Conclusions and Future Directions

The study proves the effectiveness of FL in solving complex problems in IoT security. Different ML algorithms, including KNN, LR, SVM, NB, and FL-DSFA, have shown their capacity to detect routing vulnerabilities effectively. All performance parameters, including accuracy, precision, recall and F1 score, are observed at consistently high levels for varied models, reflecting their capability to distinctly identify normal from harmful network behavior. The SVM and KNN models are notable for their exceptional performance, regularly reaching accuracy rates of approximately 95% on both the test and validation datasets. Moreover, upon comparing federated learning with conventional machine learning techniques, it becomes apparent that it has several distinct advantages, such as enhanced data confidentiality, distributed processing, and expandability. The results highlight the substantial influence that federated learning can have on improving the security of IoT deployments. This technology enables the development of more effective intrusion detection algorithms to tackle rising cyber threats. The performance findings of the FL-DSFA model also demonstrated better detection efficiency. This solution requires the modification of the firmware of IoT devices; however, its computational expense remains modest. Our upcoming research aim is to extend the detection range by utilizing a more efficient and economical calculation process.

Future studies may explore progressive global model aggregation methods to improve efficiency and convergence in the FL environment. Other advanced ML models, such as XGBoost and LightGBM, can advance identification accuracy while upholding computational efficacy. Escalating the FL-DSFA to cover a wider variety of IoT routing attacks and directing scalability in varied IoT systems can be explored. Furthermore, employing and testing this technique in real-world IoT scenarios will be critical to authenticating its efficiency and practicality under operative circumstances.

## Figures and Tables

**Table 1:** Security challenges in IoT attack mitigation studies.

Ref.	Approach	Description	Privacy
[33]	CNN ResNet model	Transformed network traffic data into images for DoS/DDoS detection	No
[34]	Radio-based RF jamming mitigation	Utilized programmable beam-steering antennas for real-time jammer categorization	No
[35]	UNSW-NB15 data analysis	Investigated flow, MQTT, and TCP feature clusters	No
[36]	Machine learning algorithms	Evaluated nine ML algorithms for IoT malware identification	No
[37]	Simulation-based approach	Generated artificial training data for SVM classifiers	No

**Table 2:** Federated learning use cases in IoT.

Ref.	IoT Use Cases	Specification of FL	Limitations
[35]	Attack defense	An FL-based attack defense network was proposed to secure industrial IoT networks	no consideration is given for the impact of latency in communication.
[39]	Attack detection	A FL model to detect security attacks in IoT	The problem of data privacy has been overlooked
[40]	Attack detection	FL-based attack detection in industry 4.0	No research work has been carried out on scalability issues
[41]	Intrusion detection	An FL-based intrusion detection model for IoT	Its performance is not considered
[42]	Intrusion detection	Intrusion detection system using FL in IoT	The performance is not validated by comparing with ML and DL approaches
[41]	Malware detection	Malware detection in Android applications using FL	The confluence of training process is overlooked
[43]	Intrusion detection	A review of FL techniques for detecting intrusion is considered	Coordination between different IoT devices is a major problem
[44]	Data breaching	FL-based identification and prevention of data breaches in industrial IoT	Larger datasets need to be tested
[45]	Malware detection in IoT devices	FL-based security enhancement in IoT	Energy performance and learning ability is overlooked

**Table 3:** Deep learning models for IoT applications.

Ref.	Approach	Description	Results
[47]	TensorFlow DNN	Identify pirated software through source code copying	97.46% classification accuracy
[48]	Deep learning framework for secure smart city	Utilize blockchain for decentralized communication in CPS	Precision: 0.7244, Recall: 0.7078, F1 score: 0.7118
[49]	FFDNN Wireless IDS with WFEU	Intrusion detection system equipped with Wireless Feature Extraction Unit	Binary classification: 87.10% accuracy, multiclass classification: 77.16% accuracy
[50]	Sequential methodology with Text-CNN and GRU	Collect network layer and application layer attributes for intrusion detection	F1 score: 0.98
[51]	Deep learning-based IDS for IoT networks	Categorize data flow for multiclass and binary classification	NSL-KSS dataset: 99.5% accuracy, CIDDS-001 dataset: 99.3% accuracy, UNSWNB15 dataset: 99.1% accuracy
[52]	IoT-IDCS-CNN	Harness convolutional neural networks for intrusion detection	Binary classification: 99.30% accuracy, multiclass classification: 98.20% accuracy

**Table 4:** Dataset description.

Attack Type	No. of Values	Size (GB)
Hello Flood Attack	64,178,435	0.75
Version Number Attack	22,868,210	0.27
Decreased Rank Attack	49,873,385	0.58

**Table 5:** Simulation data.

Metric	Length	Info	Trans Rate	Rcv Rate	TR/RR	Sources Count	Dest Count
Length	83.58	15.5	64.0	64.0	87.0	99.0	102.0
Info	3.14	1.65	1.0	2.0	3.0	5.0	7.0
Trans Rate (per 1000 ms)	0.15	0.07	0.0	0.1	0.16	0.2	0.29
Rcv Rate (per 1000 ms)	0.36	0.21	0.0	0.21	0.34	0.49	0.9
TR/RR	0.62	1.08	0.0	0.3	0.46	0.71	225.0
Sources Count/s	147.97	69.71	1.0	95.0	156.0	201.0	286.0
Destinations Count/s	361.08	208.91	1.0	209.0	345.0	493.0	901.0
Trans Total Duration/s	0.37	0.45	0.0	0.18	0.29	0.42	9.99
Rcv Total Duration/s	0.84	0.65	0.0	0.46	0.82	1.0	9.93
Trans Average/s	0.0	0.01	0.0	0.0	0.0	0.0	1.47
Rcv Average/s	0.0	0.01	0.0	0.0	0.0	0.0	1.47
DAO	28.91	66.58	0.0	0.0	0.0	0.0	286.0
DIS	51.08	78.78	0.0	0.0	0.0	145.0	260.0
DIO	10.15	29.74	0.0	0.0	0.0	0.0	227.0
Label	0.45	0.5	0.0	0.0	0.0	1.0	1.0

**Table 6:** Features and attack detection.

No.	Features	Attack Detected Min–Max (X)
1	Reception rate HF, VN	$6.6879305\times 10^{-3}$
2	Transmission rate HF, DR, and VN	$7.8469016\times 10^{5}$
3	Rcv average per Second HF, DR, and VN	$6.7000000\times 10^{0}$
4	Rcv total duration Per Second HF, DR, and VN	$1.0000000\times 10^{3}$
5	DAO DR	$9.9997274\times 10^{0}$
6	DIS DR and VN	$1.0000000\times 10^{0}$
7	Trans total duration per Second HF, VN	$3.0000000\times 10^{0}$
8	DIO HF, DR, and VN	$1.0000000\times 10^{0}$
9	TR/RR HF, DR, and VN	$4.5000000\times 10^{0}$

**Table 7:** Simulation parameters.

Parameter	Value
IDE	Google Colaboratory
Computation	GPU
Type	NVIDIA Tesla T4
RAM	12.68 GB
CUDA Version	11.2
Number of GPUs	1
Programming Language	Python 3.8
Modeling Library	TensorFlow, Scikit-learn, Keras, Seaborn
	Adam, SentenceSplitter, Pandas, Torch

**Table 8:** State of the Art Comparison.

Ref.	Dataset	Scalability	Privacy	Model Used	Attack	Findings
This Study	IRAD	Yes	Yes	FL-DSFA (LR, KNN, SVM, NB)	Hello Flood, Decreased Rank, and Version Number	SVM: Accuracy = 95%, Precision = 94%, Recall = 97%, F1 score = 96% NB: Accuracy = 89%, Precision = 99%, Recall = 79%, F1 score = 88% KNN: Accuracy = 95%, Precision = 95%, Recall = 95%, F1 score = 95% LR: Accuracy = 94%, Precision = 92%, Recall = 96%, F1 score = 94%
[54]	IRAD	No	No	LR, KNN, SVM, NB, MLP	Hello Flood, Decreased Rank, and Version Number	Accuracy = 98%, Precision = 97%, Recall = 98%, F1 score = 97%
[55]	RPL-ELIDS 783,176 (self-constructed)	No	No	ELG-IDS	internal attacks: Version Number, Decreased Rank, and DIS flooding attacks	Accuracy = 97.90%
[38]	IRAD	No	No	ANN	Decreased Rank attacks	Accuracy = 97.14%, Precision = 97.03%, Recall = 96.39%, F1 score = 96.39%
[56]	RPL-IoBT network (self-constructed)	No	No	IoBTSec-RPL	Rank, Version, blackhole, Hello Flood, and sinkhole attacks	Accuracy = 98.1%, Precision = 98.46%, Recall = 98.1%, F1 score = 96.00%
[57]	self-generated LIoTN-RPL dataset.	No	No	ProSenAD	protocol-specific rank attacks and sensor network-inherited wormhole attacks	Accuracy = 0.98%, Precision = 99%, Recall = 98.1%, F1 score = 98%
[58]	Edge Nodes (ENs) data	Yes	Yes	CFL-IDS	IIoT intrusion detection	Accuracy = 0.94%
[59]	CIC-IDS-2017	Yes	Yes	FL-SCNN-Bi-LSTM model	Wireless Sensor Networks intrusion detection	Accuracy = 0.99%

## Data Availability

Data are contained within the article.

## Abbreviations

The following abbreviations are used in this manuscript:

IoT	Internet of Things
RPL	Routing Protocol for Low-Power and Lossy Networks
FL-DSFA	Federated Learning-based Detection Mechanism for Selective Forwarding Attack
IRAD	IoT Routing Attack Dataset
HF	Hello Flood
DR	Decreased Rank
VN	Version Number
LR	Logistic regression
KNN	K-Nearest Neighbors
SVM	Support Vector Machine
NB	Naive Bayes
DoS	Denial of Service
DDoS	Distributed Denial of Service
CNN	Convolutional Neural Network
TCP	Transmission Control Protocol
MQTT	Message Queuing Telemetry Transport
RF	Random Forest
NN	Neural Networks
ST	Stacking
BG	Bagging
DT	Decision Tree
ATD	Artificial Training Data
SC-SVM	Single-Class SVM
FDA	Federated Defense Approach
IDS	Intrusion Detection System
CPS	Cyber-Physical Systems
FFDNN	Feed-Forward Deep Neural Network
WFEU	Wrapper-Based Feature Extraction Unit
GRU	Gated Recurrent Units
LSTM	Long Short-Term Memory Networks
PCAP	Packet Capture
CSV	Comma-Separated Value
LDA	Linear Discriminant Analysis
DAO	Destination Advertisement Object
DNN	Deep Neural Network
FL	Federated Learning
ANN	Artificial Neural Networks
